# The effect of side-chain substitution and hot processing on diketopyrrolopyrrole-based polymers for organic solar cells†
†Electronic supplementary information (ESI) available. See DOI: 10.1039/c7ta01740e


**DOI:** 10.1039/c7ta01740e

**Published:** 2017-06-08

**Authors:** Gaël H. L. Heintges, Pieter J. Leenaers, René A. J. Janssen

**Affiliations:** a Molecular Materials and Nanosystems , Institute for Complex Molecular Systems , Eindhoven University of Technology , P.O. Box 513 , 5600 MB Eindhoven , The Netherlands . Email: r.a.j.janssen@tue.nl; b Institute for Materials Research (IMO-IMOMEC) , Design & Synthesis of Organic Semiconductors (DSOS) , Hasselt University , Agoralaan, 3590 Diepenbeek , Belgium; c Dutch Institute for Fundamental Energy Research , De Zaale 20 , 5612 AJ Eindhoven , The Netherlands

## Abstract

The effects of cold and hot processing on the performance of polymer–fullerene solar cells are investigated for diketopyrrolopyrrole (DPP) based polymers that were specifically designed and synthesized to exhibit a strong temperature-dependent aggregation in solution. The polymers, consisting of alternating DPP and oligothiophene units, are substituted with linear and second position branched alkyl side chains. For the polymer–fullerene blends that can be processed at room temperature, hot processing does not enhance the power conversion efficiencies compared to cold processing because the increased solubility at elevated temperatures results in the formation of wider polymer fibres that reduce charge generation. Instead, hot processing seems to be advantageous when cold processing is not possible due to a limited solubility at room temperature. The resulting morphologies are consistent with a nucleation-growth mechanism for polymer fibres during drying of the films.

## Abstract 2

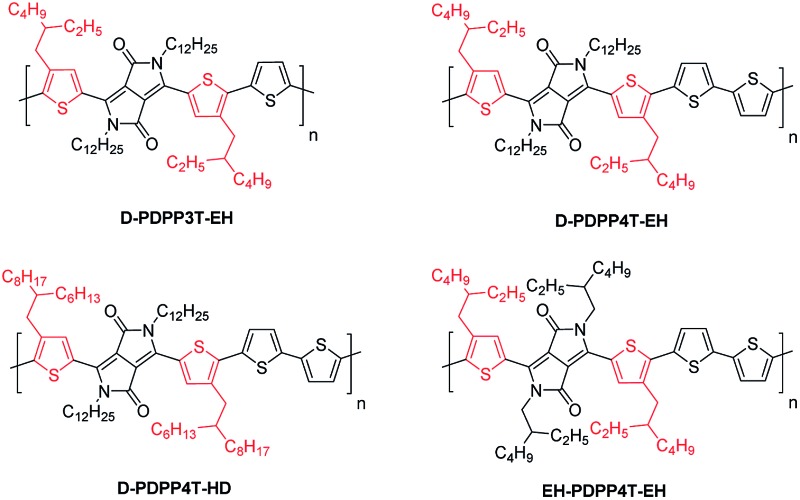
The effects of cold and hot processing on the performance of polymer–fullerene solar cells are investigated for polymers designed to exhibit temperature-dependent aggregation in solution *via* second-position branched alkyl side chains.

## Introduction

Organic photovoltaic cells have been extensively investigated as an interesting alternative photovoltaic technology, displaying the potential for semi-flexible devices produced *via* roll-to-roll methods.^1^–^3^ Typically, the active layer in these cells consists of light harvesting donor and acceptor materials. The blend morphology strongly influences the efficiency of the cell. The mechanisms of morphology formation have been studied extensively in the past. In polymer–fullerene bulk-heterojunction systems multiple methods have been used to fine-tune the morphology such as changing processing conditions, improving the molecular weight of the polymer, and adjusting the solubility by changing the solubilizing side-chains.^4^

Recently, Yan and co-workers have developed an interesting new class of semiconducting polymers that exhibit a strong temperature-dependent aggregation behaviour in solution, providing polymer–fullerene solar cells with a power conversion efficiency (PCE) of 10.8%.^5^ The strong temperature-dependent aggregation was introduced *via* alkyl side chains on the polymer backbone that have a branching point on the second carbon atom from the main chain. When these 2-alkylalkyl substituents were introduced into an oligothiophene segment in the main chain, controlled aggregation and strong crystallization of the polymer during the film cooling and drying process was achieved resulting in optimal bulk heterojunction morphologies and high PCEs under hot processing conditions.^5^ This same principle has been applied by Yan *et al.* to wide bandgap (1.90 eV),^6^ medium bandgap (1.65 eV),^5^ and low bandgap (1.43 eV)^7^ polymer donors, all reaching excellent PCEs in solar cells with fullerene acceptors. In these examples it was essential to tune the aggregation behaviour of the polymer by balancing the tendency of the π-conjugated main chain units to aggregate with the solubilizing power of the 2-alkylalkyl substituents.

Such balance has also been found to be important for morphology formation in photoactive blends based on diketopyrrolopyrrole (DPP) based π-conjugated polymers and fullerenes.^8^–^14^ In these DPP polymers, solubility in organic solvents is typically provided by solubilizing 2-alkylalkyl side chains on nitrogen atoms of the electron deficient DPP core and in relevant cases further enhanced by side chains on the heteroaromatic electron rich units of these push–pull low bandgap materials. The final morphologies are determined by the chemical structure, the processing conditions, and the molecular weight.^10^,^12^ One result that emerges from this work is that reducing the solubility, *via* either the molecular structure, increasing the molecular weight, or specific processing additive, leads to morphologies in which narrow crystalline DPP polymer fibres are present that provide high power conversion efficiencies.^9^–^14^ Under conditions where the solubility of the polymer is too high, the fibre width in the photoactive layer increases resulting in a loss in device performance because the width of the fibre exceeds the exciton diffusion length. The reasons for forming wider fibres in more soluble materials are not yet fully understood but can be rationalized with a nucleation-growth model to originate from either a minimum width of stable polymer fibres (fast-nucleation limit) or from the number of nuclei formed (fast-growth limit).^12^ Fibre formation has also been observed for other polymer–fullerene systems.^15^,^16^

Here we investigate the effects of hot *versus* cold processing of polymer–fullerene based solar cells using newly synthesized DPP-based polymers with solubilizing 2-alkylalkyl substituents on terthiophene and quaterthiophene units and linear or branched alkyl chains on the DPP unit. Changing from the common branched to a linear alkyl chain on the DPP units is expected to enhance aggregation and reduce solubility such that more narrow fibres are formed, while the branched alkyl side chains can endow the polymer with sufficient solubility at elevated temperatures.^7^ Each of the new DPP polymers exhibits a temperature-dependent aggregation. We find that for the polymers that can be processed at room temperature, hot processing does not offer an advantage in terms of device performance, because the increased processing temperature enhances the solubility and results in wider fibres.

## Results and discussion

**Scheme 1:**
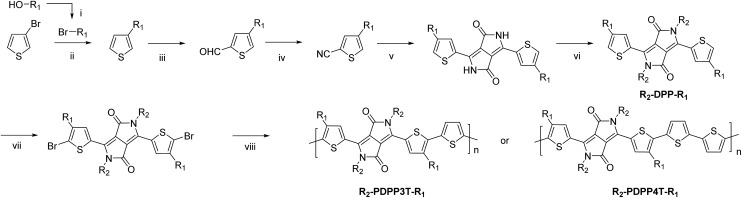
R_1_ = 2-ethylhexyl (EH) or 2-hexyldecyl (HD), R_2_ = dodecyl (D) or 2-ethylhexyl, (i) PPh_3_, NBS, DCM, 0 °C; (ii) Mg, Ni(dppp)Cl_2_, ether; (iii) (1) LDA, THF, –78 °C; (2) DMF, –78 °C; (iv) NH_2_OH·HCl, DMF, 145 °C; (v) Na, diethylsuccinate, *t*-amylalcohol, 120 °C; (vi) K_2_CO_3_, Br–R_2_, DMF, 120 °C; (vii) NBS, CHCl_3_, 0 °C; (viii) 2,5-bis(trimethylstannyl)thiophene or 5,5′-bis(trimethylstannyl)-2,2′-bithiophene, Pd_2_(dba)_3_/PPh_3_, toluene/DMF, 115 °C.

**Fig. 1:**
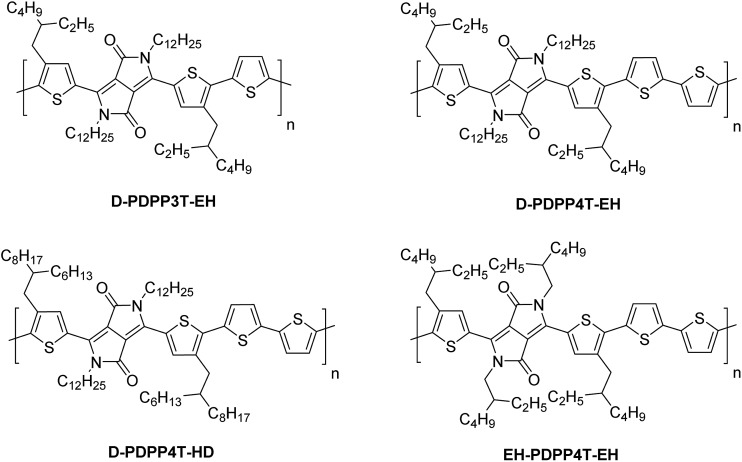
Structures of DPP polymers with branched side chains on the thiophene substituents that flank the DPP unit.

The synthesis of the monomers is shown in Scheme 1. The 2-ethylhexyl or 2-hexyldecyl side chains were introduced on the thiophene rings *via* a Kumada reaction between 3-bromothiophene and the corresponding 2-alkylalkyl magnesium bromide. Formylation and subsequent reaction with hydroxylamine hydrochloride afforded the 4-(2-alkylalkyl)-2-cyanothiophenes which were used as the starting point for the DPP formation reaction. Modified literature procedures then afforded the DPP monomers (denoted as R_2_-DPP-R_1_),^7^,^17^ which were brominated and used in a Stille polymerization reaction with either 2,5-bis(trimethylstannyl)thiophene or 5,5′-bis(trimethylstannyl)-2,2′-bithiophene under optimized conditions^18^ to afford the R_2_-PDPP3T-R_1_ and R_2_-PDPP4T-R_1_ polymers depicted in Fig. 1. The details of the synthetic procedures and characterization of the intermediates can be found in the ESI.†

The D-DPP-EH monomer was polymerized in a Stille reaction with thiophene and the resulting polymer, D-DPP3T-EH (Fig. 1), showed high solubility despite a reasonably high molecular weight of *M*_n_ = 39 kDa (Table 1). We attribute the high solubility to the two branched side chains on the terthiophene moieties that separate the DPP units along the chain. As we know that a too high solubility at room temperature of these polymers causes a reduction of the solar cell performance by forming a too coarse phase separation,^9^–^12^ we reduced the solubility by introducing an additional unsubstituted thiophene ring in the main chain *via* the polymerization of D-DPP-EH and bithiophene. In the resulting D-PDPP4T-EH (Fig. 1) polymer, the alkyl substituents are more distant and point in opposite directions along the thiophene chain. The additional thiophene ring in the repeating unit had a dramatic effect and resulted in a significant decrease in solubility. The rather low *M*_n_ of 24.3 kDa of D-PDPP4T-EH can be explained by precipitation of the material during the reaction before polymerization was completed. To achieve a solubility in between those of D-PDPP3T-EH and D-PDPP4T-EH, two additional polymers were synthesized (Fig. 1). D-PDPP4T-HD features extended 2-hexyldecyl side chains on the thiophenes flanking the DPP unit, while in EH-PDPP4T-EH the linear dodecyl chain on the DPP unit is replaced by a branched 2-ethylhexyl chain. A rather high *M*_n_ of 57.3 kDa was obtained for D-PDPP4T-HD, indicating that it has an improved solubility in the polymerization reaction yielding a higher molecular weight. The *M*_n_ of EH-PDPP4T-EH could, however, not be determined by GPC as it showed a remarkably very low solubility in *o*-dichlorobenzene (*o*-DCB) even at 140 °C (see the ESI†).

**Table 1:** Physical properties of the polymers

Polymer	*M*_n_ [kDa]	*M*_w_ [kDa]	PDI	*E*_g_ ^*a*^ [eV]	HOMO^*b*^ [eV]	LUMO^*b*^ [eV]	*E*_g_,^*c*^ [eV]	Ref.
HD-PDPP3T	147	400	2.72	1.35	–5.60	–3.76	1.84	18
DT-PDPP3T	137	458	3.34	1.36	—	—		9
HD-PDPP3T-Me	110	282	2.56	1.34	–5.61	–3.71	1.90	17
D-PDPP3T-EH	39.0	90.0	2.31	1.35	–5.60	–3.66	1.94	This work
DT-PDPP4T	219	641	2.93	1.43	–5.59	–3.65	1.94	9
EH-PDPP4T-HD	—	—	—	1.45	—	—		7
D-PDPP4T-EH	24.3	59.3	2.44	1.43	–5.54	–3.69	1.85	This work
D-PDPP4T-HD	57.3	173	3.02	1.41	–5.59	–3.58	2.01	This work
EH-PDPP4T-EH	—	—	—	1.48	–5.58	–3.65	1.93	This work

^*a*^From the absorption onset in thin films.

^*b*^From the onsets of the redox waves and using –5.23 eV for the energy of ferrocene/ferrocenium.

^*c*^Electrochemical bandgap.

**Fig. 2:**
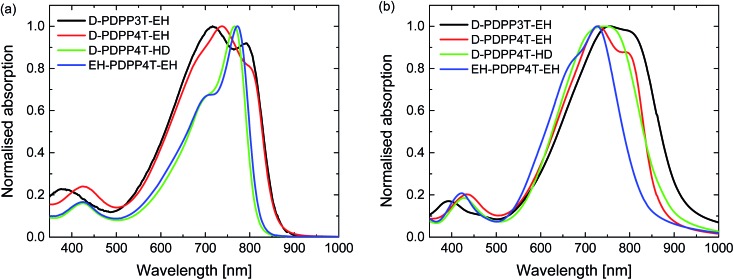
Normalized UV-vis-NIR absorption spectra of the DPP polymers. (a) In chloroform solution. (b) As thin film on glass.

UV-vis-NIR absorption spectra of the materials measured in chloroform and thin films (Fig. 2 and S1, ESI†) reveal an interesting behaviour. For D-PDPP3T-EH there is a significant red shift in the optical bandgap when going from solution to thin film. While it is common for many conjugated polymers to exhibit such shift, the effect is larger for D-PDPP3T-EH than for the previously studied polymers HD-PDPP3T^18^ and HD-PDDP3T-Me^17^ that have no or a small (–CH_3_) substituent on the thiophene rings flanking the DPP unit. The shift of the onsets of absorption in solution and film, determined from the crossing point of the tangent in the inflection point in the spectrum at the long wavelength with the horizontal axis, decreases from 58 nm for D-PDPP3T-EH to 48 nm for DT-PDPP3T, 36 nm for HD-PDDP3T-Me, and 12 nm for HD-PDPP3T (Fig. S2, ESI†). This supports the notion that in solution D-PDPP3T-EH is less aggregated due to the branched side chains on the thiophene rings. The higher solubility is associated with twisting of the polymer chain, which reduces the effective conjugation length. In the solid state, the optical bandgap of all three PDPP3T derivatives is very similar and amounts to *E*_g_ = 1.34 eV. As we will demonstrate in greater detail below, the vibronic peaks in the spectrum of D-PDPP3T-EH are associated with its partial aggregation under these conditions.

The PDPP4T polymers behave quite differently. D-PDPP4T-EH shows a rather low bandgap in solution and the absorption is not shifted in the solid state (Fig. 2). This behaviour is similar to that of DT-PDPP4T (DT = 2-decyltetradecyl) which also did not show a shift in the onset of the absorption between solution and thin film spectra.^9^ The two more soluble D-PDPP4T-HD and EH-PDPP4T-EH polymers do have significantly blue-shifted onsets of absorption in solution compared to D-DPP4T-EH (Fig. 2). This suggests that they are less aggregated, but we note that they show a clear vibronic shoulder at higher energies that often appears when interchain interactions are also present. Of these two more soluble derivatives, D-PDPP4T-HD exhibits a pronounced red shift of its thin film absorption spectrum, to an onset that is similar to that of DT-PDPP4T and D-PDPP4T-EH (Fig. 2, Table 1). Remarkably, for EH-PDPP4T-EH the absorption onset does not shift significantly between solution and solid states. This suggests that upon casting a film of EH-PDPP4T-EH the chains do not undergo more aggregation.

Cyclic voltammetry was performed on polymer films cast on indium tin oxide (ITO) glass substrates in acetonitrile containing tetrabutylammonium hexafluorophosphate as the electrolyte (Fig. S3 and Table S1, ESI†). The HOMO and LUMO levels determined from the onsets of the oxidation and reduction waves vary little, suggesting a small effect of the alkyl chain substitution pattern on the energy levels of the polymer (Table 1). In each case, the electrochemical bandgap is significantly larger than the optical bandgap.

**Fig. 3:**
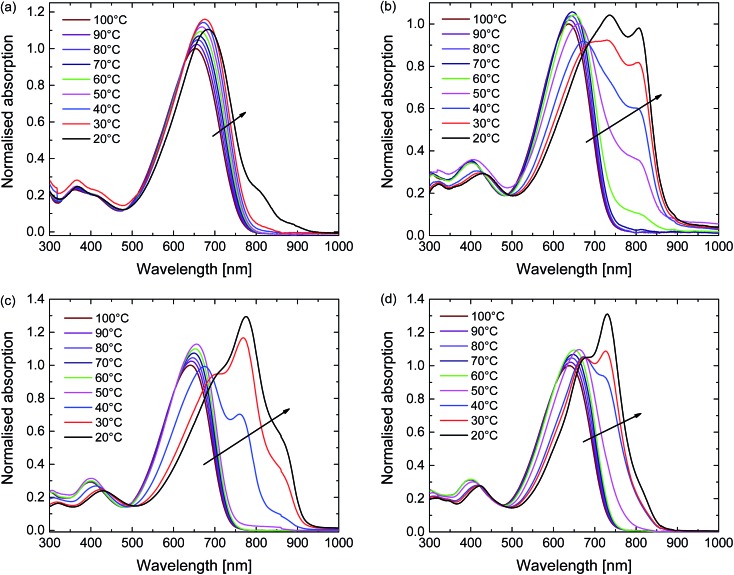
Temperature-dependent UV-vis-NIR absorption of the polymers in TCE solution. (a) D-PDPP3T-EH. (b) D-PDPP4T-EH. (c) D-PDPP4T-HD. (d) EH-PDPP4T-EH.

The aggregation behaviour of the polymers was further explored using temperature-dependent UV-vis-NIR measurements in 1,1,2,2-tetrachloroethane (TCE) (Fig. 3). TCE is a slightly less effective solvent for DPP polymers compared to chloroform, but its higher boiling point (147 °C *vs.* 61 °C) allows the study of solutions over a much wider temperature range. At the highest temperature used (100 °C) each polymer is truly molecularly dissolved. At this stage the spectra show a single unresolved absorption band centred at ∼650 nm (Fig. 3). With decreasing temperature the UV-vis-NIR spectra of all four polymers in TCE show a transition from the molecularly dissolved state to an aggregated state, which is evidenced from the appearance of a long wavelength peak and the associated vibronic structure. This transition starts at ∼60 °C for D-PDPP4T-EH and at ∼50 °C for D-PDPP4T-HD and EH-PDPP4T-EH, while it only occurs at ∼20 °C for D-PDPP3T-EH, which is the most soluble polymer of the four derivatives. For the three PDPP4T polymers further cooling below the onset of aggregation is accompanied by an isosbestic point in the spectra, which we interpret as the transition between an aggregated and a molecularly dissolved state. Above the transition temperature, where no aggregates exist, there is no isosbestic point. In this regime the absorption maxima simply shift gradually to lower energy when reducing the temperature. This is attributed to a gradual decrease of the twisting of the polymer chains. This planarization increases the average effective conjugation length of chain segments and moves the absorption maximum to lower energies. The small increase in intensity at lower temperatures is, at least partly, due to thermal contraction of the solvent. In the ESI we show (Fig. S4†) and discuss the variable temperature spectra of the four polymers in *o*-DCB, which is a less effective solvent than TCE.

Solar cell devices were made using the polymers as the electron donor in combination with [6,6]-phenyl-C_71_-butylric acid methyl ester ([70]PCBM) as the electron acceptor on glass substrates patterned with ITO, covered with poly(3,4-ethylenedioxythiophene)-poly(styrenesulfonate) (PEDOT:PSS) as the hole extracting contact, and LiF/Al as the electron extracting contact. The processing conditions for the active layers were carefully optimized for each individual polymer using *o*-DCB, diphenyl ether (DPE), or 1,8-diiodooctane (DIO) as the co-solvent where appropriate. We evaluated both room temperature (20 °C from chloroform (CF)) and hot (100 to 140 °C from TCE) deposition conditions.

**Fig. 4:**
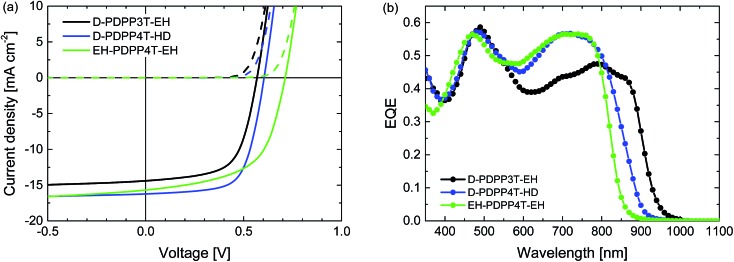
Characteristics of the optimized R_2_-PDPP-R_1_:[70]PCBM solar cells processed at room temperature from CF/DPE (98 : 2). (a) *J*–*V* characteristics under white light illumination. (b) EQE.

Room temperature processing was performed using chloroform as the main solvent with *o*-DCB, DPE or DIO as the co-solvent. Because D-PDPP4T-EH is not sufficiently soluble in chloroform at room temperature, the analysis was limited to the three more soluble polymers. Table S2 (ESI†) shows that using DPE as the co-solvent affords higher PCEs than the use of *o*-DCB or DIO. The current density–voltage (*J*–*V*) characteristics and external quantum efficiency (EQE) of the devices processed at room temperature from chloroform/DPE (98 : 2) are shown in Fig. 4 and the relevant solar cell parameters are displayed in Table 2.

**Table 2:** Processing conditions and device characteristics of the R_2_-PDPP-R_1_:[70]PCBM solar cells

Polymer	Solution	*T* [°C]	*μ*_h_ [cm^2^ V^–1^ s^–1^]	*d* [nm]	*J*_sc_ [mA cm^–2^]	*V*_oc_ [V]	FF	PCE [%]
D-PDPP3T-EH	CF/DPE (98 : 2)	20	6.8 × 10^–4^	154	15.2	0.57	0.63	5.4
	TCE	120	1.9 × 10^–3^	140	11.3	0.70	0.55	4.4
	TCE/DPE (98 : 2)	120	5.9 × 10^–4^	135	10.4	0.59	0.62	3.8
D-PDPP4T-EH	TCE/DPE (98 : 2)	120	6.5 × 10^–4^	121	10.1	0.59	0.57	3.4
D-PDPP4T-HD	CF/DPE (98 : 2)	20	6.6 × 10^–4^	101	15.8	0.60	0.67	6.3
	TCE/DIO (98 : 2)	100	1.0 × 10^–3^	88	11.9	0.61	0.70	5.1
	TCE/DPE (98 : 2)	120	8.3 × 10^–4^	147	12.1	0.63	0.60	4.5
EH-PDPP4T-EH	CF/DPE (98 : 2)	20	1.4 × 10^–3^	106	14.9	0.71	0.58	6.1
	TCE/*o*-DCB (9 : 1)	140	1.2 × 10^–3^	96	10.1	0.75	0.66	5.0
	TCE/DPE (98 : 2)	120	8.1 × 10^–4^	92	11.4	0.74	0.59	4.9

The PCE of the D-PDPP3T-EH:[70]PCBM cells reaches 5.4%. The open-circuit voltage (*V*_oc_) of this cell (0.57 V) is lower than that of the related HD-PDPP3T:[70]PCBM (*V*_oc_ = 0.67 V) cell,^18^ but almost identical to that of HD-PDPP3T-Me:[70]PCBM (*V*_oc_ = 0.59 V) cells.^17^ This difference can be attributed to the inductive electron donating effect of the alkyl substituents on the thiophene rings which raises the HOMO level and lowers the *V*_oc_. We note, however, that these rather subtle effects on the HOMO energy do not show up in the oxidation potentials determined by cyclic voltammetry. Compared to HD-PDPP3T and HD-PDPP3T-Me the EQE and, hence, the short-circuit current density (*J*_sc_) of the cells based on D-PDPP3T-EH are lower, resulting in a lower overall PCE (5.4% *vs.* 7.1% (ref. 18) and 6.8% (ref. 17)). For all three PDPP3T polymers the onset of the EQE is found at about 950 nm.

D-PDPP4T-HD and EH-PDPP4T-EH reach comparable PCEs (6.3% and 6.1%) in solar cells when blended with [70]PCBM. These values are only slightly lower than the PCE = 7.1% for DT-PDPP4T:[70]PCBM cells studied previously.^9^ For the D-PDPP4T-HD cell the main difference is in the *V*_oc_ = 0.60 V, which shows the expected decrease compared to the *V*_oc_ = 0.64 V of DT-PDPP4T as a result of the electron donating alkyl substituents on the main chain. The onsets of the EQEs for D-PDPP4T-HD and DT-PDPP4T cells are both at about 900 nm. For EH-DPP4T-EH, on the other hand, the solar cell characteristics are quite different despite the similar polymer backbone. A significantly increased *V*_oc_ = 0.71 V is found for EH-PDPP4T-EH cells, which is in accordance with the higher optical bandgap. The fill factor (FF) of the EH-PDPP4T-EH cell is lower as a result of field-dependent charge extraction, which can be seen from the significant slope of the *J*–*V* curve under short circuit conditions. This slope is not present in the dark *J*–*V* curve and hence not simply due to a low shunt (parallel) resistance.

**Fig. 5:**
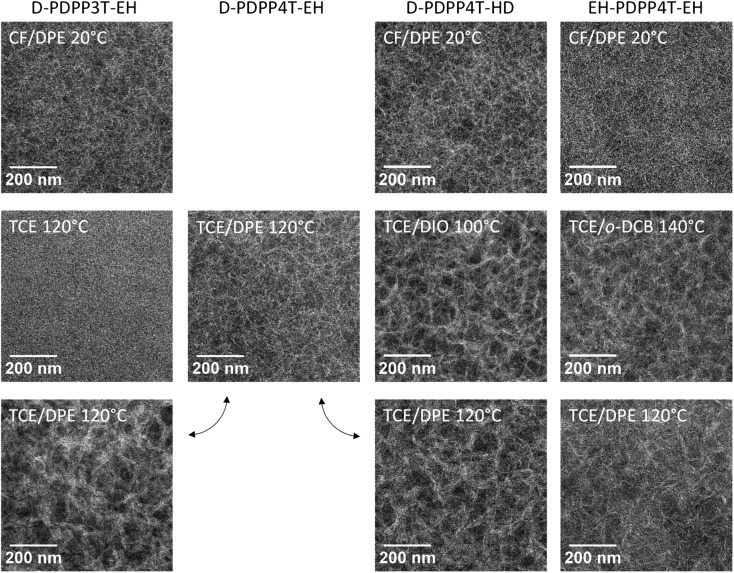
TEM images of the photoactive layers. Left to right: blends of D-PDPP3T-EH, D-PDPP4T-EH, D-PDPP4T-HD, and EH-PDPP4T-EH with [70]PCBM. Top row: room temperature processing from chloroform/DPE (98 : 2); middle row: optimized hot-processed devices from TCE/co-solvent; bottom row: hot-processed devices from TCE/DPE (98 : 2).

The morphology of the three photoactive layers was investigated by transmission electron microscopy (TEM) (Fig. 5). In each case a fibre-like morphology is observed. These fibres appear brighter and originate from semi-crystalline aggregates of the DPP polymers.^9^–^12^

A slightly coarser morphology, with wider fibres, is observed for the D-PDPP3T-EH:[70]PCBM film compared to the EH-PDPP4T-EH:[70]PCBM film (Fig. 5, top row). The wider fibres are in accordance with the higher solubility of this polymer and rationalize the reduced EQE (45%) in the wavelength region above 700 nm where only the polymer absorbs light.^9^–^12^ As expected, the higher EQE of the cells based on EH-PDPP4T-EH is associated with narrower fibres in TEM. However, the finer morphology of the EH-PDPP4T-EH-based layer compared to D-PDPP4T-HD is not clearly reflected in the EQE. Fig. 4a shows that the photocurrent in the EH-PDPP4T-EH-based blend is strongly field dependent, while that of D-PDPP4T-HD saturates quicker below *V*_oc_. This is consistent with the more intimate morphology of the EH-PDPP4T-EH:[70]PCBM blend which can hinder charge extraction due to longer or non-continuous pathways for charges, causing more non-geminate charge recombination, a bias voltage just below *V*_oc_ and a reduced fill factor. The difference in EQE for the D-PDPP3T-EH and D-PDPP4T-HD based cells cannot be readily explained by the fibre width, which is comparable, and must be determined by other factors.

**Fig. 6:**
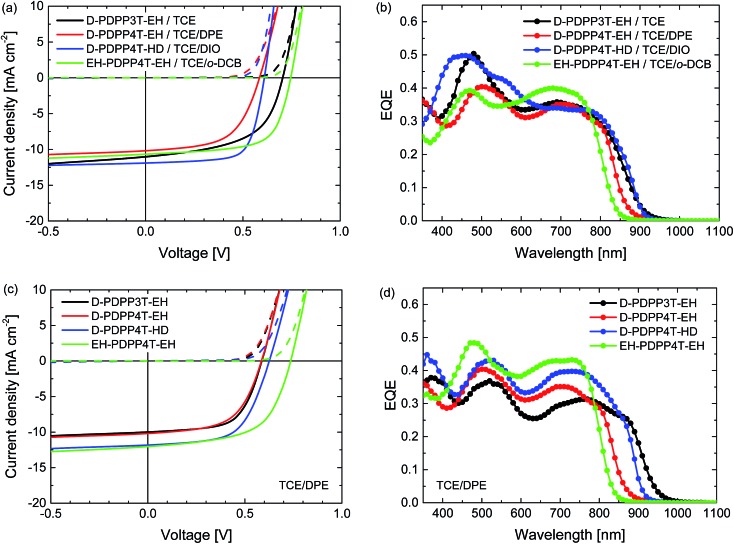
Characteristics of the optimized R_2_-PDPP-R_1_:[70]PCBM solar cells processed at 120 °C from TCE/co-solvent. (a, c) *J*–*V* characteristics under white light illumination. (b, d) EQE. (a, b) Best devices from solvent/co-solvent combination indicated in the legends. (c, d) Devices processed from TCE/DPE.

Hot processing of the active layers was performed to take advantage of the temperature-dependent aggregation behaviour of the polymers. We used TCE as the main solvent and solutions were heated to 100, 120, or 140 °C. Again we tested *o*-DCB, DPE and DIO as the co-solvent, after using pure TCE. The results of the optimisation are displayed in Table S3 (ESI†). For hot processing the co-solvent that affords the highest PCE varies among the different polymers, but in each case TCE/DPE (98 : 2) provides a PCE that is comparable to that of the best device. In Fig. 6 and Table 2 we have included the best performing hot-processed solar cells, but also included the TCE/DPE-processed devices, such that a direct comparison under identical conditions can also be made.

The *J*–*V* characteristics and EQE data shown in Fig. 6 and Table 2 reveal that hot processing of these active layers from TCE/co-solvent does not improve the device performance compared to the room temperature processing from chloroform/DPE. The EQE and concomitantly the *J*_sc_ of the hot processed cells are lower, while the *V*_oc_ remains the same or even slightly increases, except in the case of D-PDPP3T-EH.

The difference in *V*_oc_ in the case of D-PDPP3T-EH cells processed from pure TCE can be explained by a lack of aggregation, which is also evidenced by a shift of the bandgap visible in the EQE of these cells, as well as TEM analysis. This indicates that the good solubility of D-PDPP3T-EH in TCE (Fig. 3) prevents aggregation while spin coating, which is achieved when using a co-solvent.

Compared to room temperature processing, the FF of the optimised cells decreases in the case of D-PDPP3T-EH, but increases for D-PDPP4T-HD and EH-PDPP4T-EH. To investigate these differences in the FF further, hole-only devices were fabricated using ITO/PEDOT:PSS and MoO_3_/Ag electrodes with active layers processed under identical conditions to determine the hole mobility from the space charge limited current. The hole mobilities varied in the range of 0.5 to 2.0 × 10^–3^ cm^2^ V^–1^ s^–1^ but there is no consistent correlation between the hole mobility and the fill factor (Table 2 and Fig. S5, ESI†). Bartesaghi *et al.* demonstrated that the fill factor is not only dependent on the mobility of charges,^19^ but also on the generation rate, the thickness of the device and the Langevin prefactor. The latter corrects for the reduced recombination found in solar cells compared to Langevin theory. In this case, the generation rate and thicknesses of the devices are not sufficient to explain these differences in the fill factor. The Langevin prefactor will decrease with a coarser phase separation, which will induce an increase in the fill factor. In this way, we can correlate, at least in part, the changes of the morphology (see the TEM discussion below) with these changes in the fill factor.

We note that the device performance of the D-PDPP4T-HD and EH-PDPP4T-EH hot-processed solar cells is better than that of a structurally closely related and more soluble HD-PDPP4T-HD polymer where *J*_sc_ = 9.65 mA cm^–2^, *V*_oc_ = 0.70 V, FF = 0.46, and PCE = 3.1% have been found for hot-processed cells.^7^

The TEM images of the photoactive layers (Fig. 5) demonstrate that hot-processing generally results in wider fibres and a coarser phase separation except for D-PDPP3T-EH. In fact, the TEM data enable us to rationalize the changes in *J*–*V* characteristics consistently. For D-PDPP3T-EH:[70]PCBM cells, hot processing from pure TCE results in a very homogeneous morphology without visible phase separation. For the blend processed from hot TCE the onset of the EQE (Fig. 6b) is blue-shifted compared to that from chloroform/DPE (Fig. 4b) and TCE/DPE (Fig. 6d). The larger bandgap, the reduced aggregation and the more intimately mixed morphology causes an increase in *V*_oc_ and a lower FF. When processed from TCE/DPE, the polymer does aggregate during spin coating, but forms clearly broader fibres than those during room temperature processing from CF/DPE. The wider fibres reduce exciton dissociation and charge generation, explaining the loss in *J*_sc_. Also for D-PDPP4T-HD:[70]PCBM and EH-PDPP4T-EH:[70]PCBM the hot-processed layers show wider fibres than the room-temperature processed layers, irrespective of the co-solvent used (Fig. 5). This explains the loss of photocurrent, as the width of the fibres becomes larger than the exciton diffusion length, such that not all photoexcitations result in charge formation. This behaviour is common to many DPP polymer–fullerene blends.^9^–^12^ In these cases the cells are limited by charge generation. The wider fibres in the D-PDPP4T-HD:[70]PCBM and EH-PDPP4T-EH:[70]PCBM photoactive layers do, however, improve the charge collection, likely *via* a reduced bimolecular (Langevin) recombination because charges are confined to larger domains as evidenced by the increase of the fill factors.

To understand these results it is sufficient to realize that the most important effect of raising the temperature is the increase in solubility. In this case, the width of the fibres is determined by the solubility, with wider fibres being formed when the solubility is higher.^9^–^12^ This is clearly seen in the TEM analysis, as hot processing results in wider fibres than cold processing in all cases except for D-PDPP3T-EH cast from pure TCE, where polymer aggregation is completely suppressed. The fibre width in these blends can be rationalized with a nucleation-growth model. In this model, high solubility results in wider fibres because either the critical nucleus size is larger for more soluble polymers, or the size is determined by fast growth of an initial amount of nuclei formed.^12^

It must be noted that the increased solubility and formation of larger fibres could also be caused by using TCE as the main solvent. To investigate this, attempts were made to produce devices from TCE solutions at room temperature; however gelation of the solution prevented casting of a closed layer. Instead, chloroform-based solutions were heated to 40 °C and 60 °C and solar cell devices were made at these temperatures (Table S4, ESI†). In all cases the *J*_sc_ and PCE of the devices remained similar when comparing devices fabricated at room temperature to devices made at 40 °C, but clearly decreased when going to 60 °C. This result demonstrates that hot processing in the case of these materials does not offer an advantage over cold processing.

In the discussion above we have related the fibre width and morphology to the solubility of the polymer in the solvent/co-solvent combination. In principle, the volatility of the solvents can also influence the width of the fibres and the film morphology. Chloroform is a low-boiling solvent, and when spin-cast, the active layer may dry more rapidly, inducing smaller fibres than when using (hot) TCE. Indeed the volatility of the solvent determines how fast the drying line in the ternary phase diagram of the solvent, fullerene, and polymer is traversed. Although this may have an effect on the fibre width, we demonstrated in a recent kinetic study on a related DPP-based polymer that the drying rate does not significantly affect the fibre width, in contrast to solubility.^12^ Also in the examples presented in this work, the changing solubility provides a consistent explanation for the observations.

## Conclusion

Four new semiconducting polymers were designed and synthesized to investigate the effects of cold and hot processing on the performance of polymer–fullerene solar cells. The polymers comprise electron-deficient DPP and electron-rich oligothiophenes that are substituted with linear and branched alkyl side chains to create a strong temperature-dependent solubility. All polymers show a strong temperature-dependent aggregation behaviour in solution as evidenced from temperature-dependent UV-vis-NIR absorption spectra. In thin solid films, the side chains induce significant differences in the packing and interchain interactions causing the optical absorption spectra to differ significantly even for polymers with the same conjugated backbone. Hot processing of these materials into polymer–fullerene bulk heterojunctions does not lead to an increased performance compared to cold processing. This is attributed to the enhanced solubility of the polymers at elevated temperatures, which results in coarser morphologies with wider polymer fibres. This can be rationalized with a nucleation-growth mechanism that was recently proposed.^12^ Of the studied polymers, D-PDPP4T-HD provided the highest PCE of 6.3% in combination with [70]PCBM when cold-processed from chloroform with 2% DPE as the processing additive. Hot processing (100 °C) of the same blend from TCE with 2% DIO resulted in a PCE of 5%. For these DPP polymers, hot processing seems to be advantageous only when cold processing is not possible due to a too limited solubility.

## Supplementary Material

Supplementary information
