# Supplementary material for: The effect of side-chain substitution and hot processing on diketopyrrolopyrrole-based polymers for organic solar cells
Source: J Mater Chem A Mater. 2017 Jun 8;5(26):13748–56. doi: 10.1039/c7ta01740e (PMC5735362; doi:10.1039/c7ta01740e)
Supplement: Supplementary file 1 [file TA-005-C7TA01740E-s001.pdf]

This journal is © The Royal Society of Chemistry 2017

Electronic Supplementary Information

The effect of side-chain substitution and hot processing on diketopyrrolopyrrole-based polymers for organic solar cells

Gaël H. L. Heintges, Pieter J. Leenaers, René A. J. Janssen

1. Materials and methods

All synthetic procedures were performed under an argon atmosphere. Commercial (dry) solvents and reactants were used without further purification, unless stated otherwise. Diethyl ether was dried over a column containing 4 Å molecular sieves. *N*-bromosuccinimide (NBS) was recrystallized from deionized water prior to use. 2,5-Bis(trimethylstannyl)thiophene (**9**), bis(trimethylstannyl)-2,2′-bithiophene (**10**), and triphenylphosphine (PPh_3) were recrystallized from methanol prior to polymerization. 2-Ethylhexylbromide (**2a**) was purchased from TCI Europe N.V. Dichloro(1,3-bis(diphenylphosphino)propane)nickel (Ni(dppp)Cl_2) and tris(dibenzylideneacetone)dipalladium ($Pd_2(dba)_3$) were purchased from Strem Chemicals Inc. [70]PCBM (purity 90-95%) was purchased from Solenne BV. All other chemicals and solvents were obtained from Sigma-Aldrich Co.

Fourier transform infrared spectroscopy (FTIR) was performed on a PerkinElmer Spectrum Two spectrometer in ATR mode. ^{1}H-NMR and ^{13}C-NMR spectra were recorded on a Varian Mercury (^{1}H 400 MHz or 200 MHz, ^{13}C 100 MHz) spectrometer. Chemical shifts are given in ppm with respect to tetramethylsilane as internal standard. The GC-MS system consisted of a Shimadzu (GC-2010) gas chromatograph and a Shimadzu (GCMS-QP2010plus) gas chromatograph mass spectrometer. The gas chromatograph contained a 30 meter Phenomenex Zebron ZB-5MS column with an internal diameter of 0.25 mm and a 0.25 µm stationary phase film thickness. Matrix assisted laser desorption ionization time of flight (MALDI-TOF) mass spectrometry was measured on a Bruker Autoflex Speed spectrometer. Polymer molecular-weight distributions were estimated by GPC at 140 °C on a PL-GPC 120 system using a PL-GEL 10 mm MIXED-C column with *o*-DCB as the eluent and using polystyrene internal standards. Samples were first dissolved in *o*-DCB at 140 °C for 1 hour and filtered hot through a heated 2 µm PTFE filter.

UV-vis-NIR absorption spectroscopy was conducted on a PerkinElmer Lambda 1050 spectrophotometer at room temperature. Room temperature solution spectra were recorded in $CHCl_3$. Temperature-dependent spectra were recorded in *o*-DCB and TCE at temperatures between 20 °C and 100 °C using a PerkinElmer Peltier temperature controller. The polymer films were prepared by spin coating a polymer solution (6 mg mL^{-1}) in $CHCl_3$:*o*-DCB (9:1 v/v) (for D-PDPP3T-EH, D-PDPP4T-HD, and EH-PDPP4T-EH) or pure 1,1,2,2-tetrachloroethane (TCE) (for D-PDDP4T-HD) on glass substrates at 1500 rpm. The glass substrates were cleaned with acetone and isopropanol and threated with UV-ozone for 30 min. prior to use.

Cyclic voltammetry was performed on polymer films under an inert atmosphere with a scan speed of 0.2 Vs^{-1} using a solution of 1 M tetrabutylammonium hexafluorophosphate in acetonitrile. A polymer-covered ITO substrate was used as the working electrode, a silver rod

as counter electrode, and a silver rod coated with silver chloride (Ag/AgCl) as quasi-reference electrode in combination with Fc/Fc^+ as an internal standard.

Transmission electron microscopy (TEM) was performed on a Tecnai G2 Sphera transmission electron microscope (FEI) operating at 200 kV.

2. Solar cell and hole-only devices fabrication and characterization

Photovoltaic devices with an active area of 0.09 and 0.16 cm^2 were fabricated in air on patterned indium tin oxide (ITO) glass substrates (Naranjo Substrates). The substrates were cleaned by sonication in acetone for 15 min., followed by scrubbing with a sodium dodecyl sulfate solution (99%, Acros), rinsing with deionized water, and a final sonication step in 2-propanol. Before deposition of the device layers the substrates underwent a 30 min. UV-ozone treatment. Poly(ethylenedioxythiophene):poly(styrenesulfonate) (PEDOT:PSS) (Clevios P, VP Al4083) was spin coated at 3000 rpm to form a 40 nm layer.

For the room-temperature (20 °C) processed devices, the polymers (D-PDPP3T-EH, D-PDPP4T-HD, and EH-PDPP4T-EH**)** were mixed with [70]PCBM in a 1:2 weight ratio and dissolved in $CHCl_3$ (at concentrations of 6, 5, and 6 mg mL^{-1}, respectively). The solutions were heated to 90 °C for 1 h and cooled to room temperature prior to spin coating. The best photovoltaic devices for D-PDPP3T-EH were fabricated by spin coating using 10 vol.-% *o*-DCB as processing additive at 1500 rpm. The best photovoltaic devices for D-PDPP4T-HD and EH-PDPP4T-EH were fabricated by spin coating using 2 vol.-% diphenyl ether (DPE) as processing additive at 2000 and 3000 rpm, respectively.

For the hot processed devices the D-PDPP3T-EH, D-PDPP4T-EH, D-PDPP4T-HD, and EH-PDPP4T-EH polymers were mixed with [70]PCBM in a 1:2 weight ratio and dissolved in TCE at a concentration of 10, 10, 6, and 6 mg mL^{-1}, respectively. Optimal processing temperatures varied between 100 and 140 °C (see Table 2, main text). The solution, pipettes, and substrates were heated to the required temperature between 100 and 140 °C for 1 h prior to spin coating. For D-PDPP3T-EH the best photovoltaic devices were fabricated by spin coating at 120 °C using 10 vol.-% *o*-DCB as processing additive at 3000 rpm. For D-PDPP4T-EH no processing additive was used, for D-PDPP4T-HD 2 vol.-% of DIO was optimal, and for EH-PDPP4T-EH 10 vol.-% of *o*-DCB was used. Optimal active layer thicknesses were obtained by spin coating the polymer solutions at spin speeds between 1500 and 2500 rpm.

The 1 nm LiF and 100 nm Al back electrode layers were deposited by thermal evaporation under high vacuum (~3×10^{-7} mbar).

Current density – voltage ($J-V$) characteristics were measured with a Keithley 2400 source meter under ~100 mW cm^{-2} white light illumination from a tungsten-halogen lamp filtered by a Schott GG385 UV filter and a Hoya LB120 daylight filter. The accurate short-circuit current density (J_{sc}) was determined from external quantum efficiency (EQE) measurements by integration of the EQE with the AM 1.5G solar spectrum. EQE measurements were conducted under 1 sun operating conditions in a homebuilt setup consisting of a modulated monochromatic light, a preamplifier (Stanford Research Systems SR570) and a lock-in amplifier (Stanford Research Systems SR830). The modulated monochromatic light was generated by using an optical chopper from Stanford Research Systems (SR540), an Oriel Cornerstone 130 monochromator and a 50 W (Osram 64610)

tungsten-halogen lamp. The 1 sun conditions were provided by the use of a 730 nm LED (Thorlabs) at different intensities for appropriate bias illumination. A calibrated silicon cell was used as reference prior to the $J-V$ and EQE measurements. Thermal evaporation of the back electrode and $J-V$ measurements were both performed inside a nitrogen filled glove-box. For the EQE measurements, the photovoltaic devices were encapsulated in a nitrogen filled box with a quartz window. The active layer thickness was determined on a Veeco Dektak150 profilometer.

Hole-only devices with an active area of 0.09 and 0.16 cm^2 were fabricated in air on patterned indium tin oxide (ITO) glass substrates (Naranjo Substrates). The substrates were cleaned with the same procedure as mentioned before for the solar cell devices. Poly(ethylenedioxythiophene):poly(styrenesulfonate) (PEDOT:PSS) (Clevios P, VP Al4083) was then spin coated at 3000 rpm to form a 40 nm layer. The active layers were then cast under the same conditions as for the solar cell devices. By varying the spin speed, thicknesses from 120 to 250 nm were obtained. 10 nm of MoO_3 and 100 nm of Ag were then deposited by thermal evaporation under high vacuum (~3 × 10^{-7} mbar). Current density – voltage ($J-V$) characteristics were measured with a Keithley 2400 source meter, after illumination of the cell with UV light to dope the MoO_3 for at least 10 minutes, sweeping from 0 to 6 V. For each type of active layer, three layers of different thicknesses were cast and measured. No thickness dependence of the mobility was observed. The mobility was determined by fitting the $J-V$ curves with the Mott-Gurney square law, using an empirical series resistance correction, which varied between 5 and 20 Ω. The published hole mobility is the average of 12 individual devices using three active layer thicknesses.

3. Synthesis

Scheme S1 shows the synthesis route to the four DPP polymers. In the subsequent sections the synthetic procedures and molecular characterization are provided.

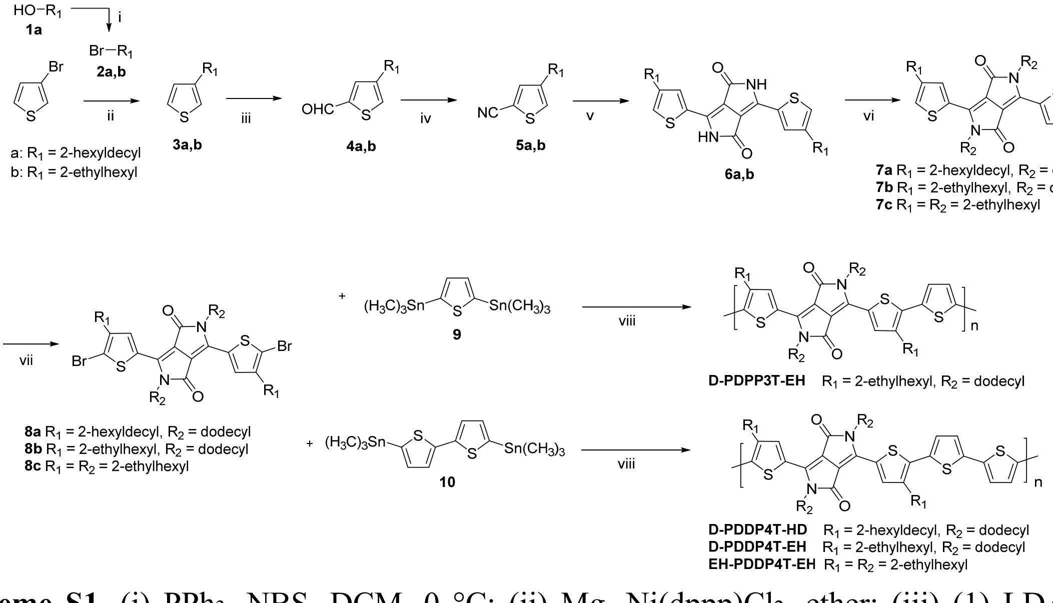

Scheme S1. (i) PPh_3, NBS, DCM, 0 °C; (ii) Mg, Ni(dppp)Cl_2, ether; (iii) (1) LDA, THF, −78 °C; (2) DMF, −78 °C; (iv) $NH_2OH.HCl$, DMF, 145 °C; (v) Na, diethylsuccinate, *t*-amylalcohol, 120 ºC; (vi) K_2CO_3, Br−R_2, DMF, 120 °C; (vii) NBS, $CHCl_3$, 0 °C; (viii) $Pd_2(dba)_3/PPh_3$, toluene/DMF, 115 °C.

1-Bromo-2-hexyldecane (2a)

2-Hexyldecan-1-ol (**1a**) (40.0 g, 165 mmol) was dissolved in dichloromethane (DCM) (200 mL) and cooled to 0 °C. Triphenylphosphine (47.6 g, 181 mmol) was added in several portions. Subsequently NBS (29.4 g, 165 mmol) was also added in portions at 0 °C. The reaction mixture was allowed to warm to room temperature and kept in the dark for 90 minutes. NBS (about 3 g) and PPh_3 (5 g) were added again until the reaction was found to be complete via ^{1}H-NMR. The mixture was then poured over a glass filter, washed with water and dried over magnesium sulfate. Triphenylphosphine oxide was removed by filtration after precipitation in heptane. The solvents were removed under reduced pressure and the crude oil was purified further by column chromatography using heptane as eluent. The product was obtained as a colourless oil (34.9 g, yield: 70%). ^{1}H-NMR (400 MHz, $CDCl_3$, δ): 3.44 (d, J = 4.7 Hz, 2H); 1.64-1.54 (m, 1H); 1.43-1.19 (m, 24H); 0.88 (t, J = 5.8 Hz, 6H).

3-(2-Hexyldecyl)-thiophene (3a)

A dried 3-neck flask was filled with magnesium chips (3.46 g, 142 mmol) and dry diethyl ether (19 mL). Compound **2a** (21.7 g, 71.2 mmol) of, mixed with dry diethyl ether (77 mL) was added dropwise to this mixture and subsequently heated to reflux for 2 hours. The prepared Grignard reagent was carefully transferred to a dry addition funnel and added dropwise to a mixture of 3-bromothiophene (9.67 g, 59.3 mmol) and a Ni(dppp)Cl_2 catalyst (0.482 g, 8.90×10^{-4} mol) in dry diethyl ether (58 mL). The mixture was left overnight under reflux. The reaction mixture was then quenched with hydrochloric acid and extracted with diethyl ether. The combined organic layers were washed with water, dried over magnesium sulfate and concentrated *in vacuo*. The remaining 3-bromothiophene was removed under high vacuum (0.2 mbar) at 50 °C and the residue was purified by column chromatography using heptane as eluent. Compound **3a** was obtained as a colourless oil (4.28 g, yield: 23%). ^{1}H-NMR (200 MHz, $CDCl_3$, δ): 7.25-7.20 (dd, J = 4.9 Hz, 3.0 Hz, 1H); 6.93-6.87 (m, 2H); 2.57 (d, J = 6.7 Hz, 2H); 1.69-1.55 (m, 1H); 1.36-1.19 (m, 24H); 0.89 (t, J = 5.7 Hz, 6H). ^{13}C-NMR (100 MHz, $CDCl_3$, δ): 141.90; 128.79; 124.75; 120.61; 38.94; 34.70; 33.33; 31.91; 30.01; 29.69; 29.63; 29.34; 26.62; 26.59; 22.69; 14.13 (Note: some peaks in the ^{13}C-NMR spectrum overlap). GC-MS: 7.57 min., m/z = 307 (2, M−1), 223 (2, $C_{14}H_{23}S^+$), 98 (100, $C_5H_6S^+$), 97 (29, $C_5H_5S^+$), 71 (10, $C_5H_{11}^+$), 57 (19, $C_4H_9^+$), 43 (21, $C_3H_7^+$).

3-(2-Ethylhexyl)-thiophene (3b)

The same procedure as for compound **3a** was employed. 1-bromo-2-ethylhexane (20.01 g, 103.6 mmol) in diethyl ether (110 mL), and magnesium chips (5.036 g, 207.2 mmol) in diethyl ether (30 mL) were used to prepared the Grignard reagents which was reacted with 3-bromothiophene (14.07 g, 86.33 mmol) using Ni(dppp)Cl_2 (0.702 g, 12.95×10^{-4} mol) as a catalyst in diethyl ether (30 mL). Compound **3b** was obtained as a colourless oil. (9.5 g, yield: 56%). ^{1}H-NMR (400 MHz, tetrachloroethane-d2, δ): 7.21-7.18 (dd, J = 4.9 Hz, 3.0 Hz, 1H); 6.90-6.86 (m, 2H); 2.52 (d, J = 6.8 Hz, 2H); 1.55-1.45 (m, 1H); 1.29-1.15 (m, 8H); 0.87-0.80 (m, 6H). ^{13}C-NMR (100 MHz, $CDCl_3$, δ): 141.89; 128.78; 124.79; 120.64; 40.42; 34.30; 32.54; 28.93; 25.65; 23.07; 14.15; 10.86. GC-MS: 4.77 min., m/z = 196 (6, M), 98 (100, $C_5H_6S^+$), 97 (52, $C_5H_5S^+$), 57 (34, $C_4H_9^+$).

4-(2-Hexyldecyl)-2-thiophenecarbaldehyde (4a)

Compound **3a** (4.12 g, 13.4 mmol) of was dissolved in dry tetrahydrofuran (THF) (20 mL). The solution was cooled to −78 °C and lithium diisopropylamide (LDA) solution in THF (2 M, 7.4 mL 14.7 mmol) was added dropwise. The reaction mixture was warmed to room temperature after 30 minutes of stirring. After 4 hours, dry *N,N*-dimethylformamide (DMF) (2.0 mL, 26.72 mmol) was added dropwise at −78 °C. Subsequently, the mixture was allowed to warm to room temperature and stirred overnight. The reaction mixture was quenched with water and extracted with diethyl ether. The combined organic layers were washed with water, dried over magnesium sulfate and concentrated under reduced pressure. The residue was purified using column chromatography (silica gel, eluent gradient 60:40 > 50:50 heptane:DCM) to give an orange oil (3.40 g, yield: 53%). ^{1}H-NMR (200 MHz, $CDCl_3$, δ): 9.86 (d, J = 1.2 Hz, 1H); 7.55 (d, J = 1.5 Hz, 1H); 7.34-7.31 (m, 1H); 2.56 (d, J = 6.8 Hz, 2H); 1.68-1.48 (m, 1H); 1.36-1.15 (m, 24H); 0.86 (t, J = 6.4 Hz, 6H). ^{13}C-NMR (100 MHz, $CDCl_3$, δ): 182.94; 143.51; 143.44; 137.64; 131.19; 38.93; 34.58; 33.18; 33.17; 31.88; 31.84; 29.93; 29.60; 29.58; 29.30; 26.56; 26.54; 22.66; 22.64; 14.09; 14.08. FTIR: $\tilde{\nu}_{max}$ [cm^{-1}]: 3087 (CH aromatic); 2955, 1378 (s) (CH_3); 2923, 2853, 1457, 722 (CH_2); 1673 (CO); 1542 (CC aromatic). GC-MS: 8.55 min., m/z = 335 (3, M−1), 126 (100, $C_6H_6OS^+$), 125 (15, $C_6H_5OS^+$), 97 (16, $C_5H_5S^+$), 71 (14, $C_5H_{11}^+$), 57 (24, $C_4H_9^+$), 43 (24, $C_3H_7^+$).

4-(2-Ethylhexyl)-2-thiophenecarbaldehyde (4b)

The same procedure as for compound **4a** was used with compound **3b** (5.01 g, 25.5 mmol) in dry THF (39 mL), LDA solution in THF (2 M, 14.0 mL, 28 mmol), and dry DMF (4.0 mL, 51.1 mmol). A yellow oil (3.8 g, yield: 66%) was obtained after column chromatography. ^{1}H-NMR (200 MHz, $CDCl_3$, δ): 9.88 (d, J = 1.2 Hz, 1H); 7.58 (d, J = 1.5 Hz, 1H); 7.36-7.33 (m, 1H); 2.58 (d, J = 6.8 Hz, 2H); 1.65-1.45 (m, 1H); 1.34-1.14 (m, 8H); 0.88 (t, J = 7.3 Hz, 6H). ^{13}C-NMR (100 MHz, $CDCl_3$, δ): 182.94; 143.50; 143.44; 137.62; 131.18; 40.35; 34.12; 32.34; 28.82; 25.49; 22.95; 14.07; 10.78. FTIR: $\tilde{\nu}_{max}$ [cm^{-1}]: 3087 (CH aromatic); 2958, 1380 (s) (CH_3); 2927, 2859, 1459 (CH_2); 1669 (CO); 1542 (CC aromatic). GC-MS: 6.17 min., m/z = 224 (6, M), 126 (100, $C_6H_6OS^+$), 125 (22, $C_6H_5OS^+$), 97 (28, $C_5H_5S^+$), 57 (47, $C_4H_9^+$).

4-(2-Hexyldecyl)-2-thiophenecarbonitrile (5a)

Water-free $NH_2OH.HCl$ (0.766 g, 11.0 mmol) was added in portions to a solution of **4a** (2.97 g, 8.82 mmol) in DMF (14 mL). Subsequently, the reaction mixture was heated to and kept for 3 hours at 145 °C. The resulting mixture was extracted with diethyl ether. The combined organic layers were washed with water, dried over magnesium sulfate and concentrated under reduced pressure. The residue was purified using column chromatography (silica gel, eluent gradient 100:0 > 98:2 heptane:ethyl acetate) to give a dark orange oil (1.96 g, yield: 67%). ^{1}H-NMR (400 MHz, tetrachloroethane-d_2, δ): 7.39 (d, J = 1.4 Hz, 1H); 7.15 (d, J = 1.3 Hz, 1H); 2.50 (d, J = 6.8 Hz, 2H); 1.57-1.48 (m, 1H); 1.29-1.11 (m, 24H); 0.84 (t, J = 6.7 Hz, 6H). ^{13}C-NMR (100 MHz, $CDCl_3$, δ): 142.89; 138.74;128.18; 114.53; 109.24; 38.88; 34.40; 33.15; 33.13; 31.87; 31.82; 29.90; 29.57; 29.55; 29.28; 26.53; 26.51; 22.65; 22.63; 14.09; 14.07. FTIR: $\tilde{\nu}_{max}$ [cm^{-1}]: 3093 (CH aromatic); 2955, 1378 (s) (CH_3); 2923, 2854, 1465, 723 (CH_2);

2217 (CN); 1539 (CC aromatic). GC-MS: 8.49 min., m/z = 333 (4, M), 123 (100, $C_6H_5NS^+$), 85 (24, $C_6H_{13}^+$), 71 (34, $C_5H_{11}^+$), 57 (49, C_4H_9+), 43 (44, $C_3H_7^+$).

4-(2-Ethylhexyl)-2-thiophenecarbonitrile (5b)
The same procedure as for compound **5a** was used with water-free $NH_2OH.HCl$ (1.39 g, 20.1 mmol) and **4b** (3.60 g, 16.1 mmol) in dry DMF (25 mL). An orange oil (3.04 g, yield: 85%) was obtained after column chromatography. ^{1}H-NMR (400 MHz, tetrachloroethane-d_2, δ): 7.39 (d, J = 1.3 Hz, 1H); 7.17-7.14 (m, 1H); 2.50 (d, J = 6.9 Hz, 2H); 1.53-1.42 (m, 1H); 1.27-1.10 (m, 8H); 0.87-0.79 (m, 6H). ^{13}C-NMR (100 MHz, $CDCl_3$, δ): 142.87; 138.73; 128.22; 114.52; 109.23; 40.29; 33.93; 32.29; 28.78; 25.44; 22.93; 14.06; 10.74. FTIR: $\tilde{\nu}_{max}$ [cm^{-1}]: 3092 (CH aromatic); 2959, 1380 (s) (CH_3); 2927, 2859, 1460, 727 (CH_2); 2217 (CN); 1540 (CC aromatic). GC-MS: 6.11 min., m/z = 221 (5, M), 123 (100, $C_6H_5NS^+$), 57 (75, $C_4H_9^+$).

3,6-Bis(4-(2-hexyldecyl)thiophen-2-yl)-2,5-dihydropyrrolo[3,4-c]pyrrole-1,4-dione (6a)
A sodium 2-methyl-2-butoxide solution was prepared by heating elemental sodium (0.184 g, 8.01 mmol) and *tert*-amylalcohol (10 mL) in a dried 2-neck flask to 120 °C. A catalytic amount of iron(III) chloride was added to the previous solution. A solution of diethyl succinate (0.465 g, 2.67 mmol) in *tert*-amylalcohol (1.3 mL) was added dropwise to the alkaline solution and then **5a** (1.96 g, 5.88 mmol) was quickly added. The mixture was left reacting overnight until quenched with acetic acid (6 mL) and methanol (10 mL). The quenched mixture was refluxed at 85 °C for 30 min. After cooling a dark red precipitate (0.548 g) was filtered off, washed with water and methanol, and dried overnight at 50 °C *in vacuo*. The compound was used in the next reaction without further purification. ^{1}H-NMR (400 MHz, $CDCl_3$, δ): 8.05 (s, 2H); 7.86 (s, 2H); 7.18 (s, 2H); 2.61 (d, J = 6.8 Hz, 4H); 1.72-1.62 (m, 2H); 1.30-1.20 (m, 48H); 0.90-0.83 (m, 12H). MALDI-TOF-MS: [M+] calc: 748.50, found: 748.49.

3,6-Bis(4-(2-ethylhexyl)thiophen-2-yl)-2,5-dihydropyrrolo[3,4-c]pyrrole-1,4-dione (6b)
The same procedure as for compound **6a** was used. For this reaction a mixture of elemental sodium (0.409 g, 17.8 mmol) and *tert*-amylalcohol (10 mL) were heated to 120 °C. A solution of diethyl succinate (1.03 g, 5.94 mmol) in *tert*-amylalcohol (2.9 mL) was added dropwise to the alkaline solution and then **5b** (2.89 g, 13.1 mmol) was quickly added. After work-up, a black solid (1.04 g) was obtained. The compound was used in the next reaction without further purification. ^{1}H-NMR (400 MHz, 1:1 $CDCl_3$:DMSO-d_6, δ): 10.82 (s, 2H); 7.98 (s, 2H); 7.15 (s, 2H); 2.50 (m, 4H); 1.57-1.46 (m, 2H); 1.27-1.12 (m, 16H); 0.84-0.76 (m, 12H). MALDI-TOF-MS: [M+] calc: 524.25, found: 524.27.

2,5-Didodecyl-3,6-bis(4-(2-hexyldecyl)thiophen-2-yl)-2,5-dihydropyrrolo[3,4-c]pyrrole-1,4-dione (7a)
A flask was charged with **6a** (0.230 g, 3.07×10^{-4} mol), finely crushed potassium carbonate (K_2CO_3) powder (0.127 g, 9.21×10^{-4} mol), and DMF (3 mL). After heating the reaction mixture for 15 min. at 120 °C, 1-bromododecane (0.230 g, 9.21×10^{-4} mol) was added. The

mixture was stirred overnight at 120 °C, subsequently quenched with water and extracted with DCM. The combined organic phase was washed with brine, dried over magnesium sulfate and concentrated under reduced pressure. The remaining purple solid was added to a mixture of 1,4-dioxane (120 mL), HCl (2 mL) and water (2 mL) and refluxed during 1 hour. After evaporation of the solvent the crude product was further purified by column chromatography (silica gel, eluent gradient 100:0 > 70:30 heptane:DCM). Finally the pure product (104 mg, yield: 31%) of was obtained as purple flakes after recrystallization in ethanol. ^{1}H-NMR (200 MHz, $CDCl_3$, δ): 8.70 (d, J = 1.3 Hz, 2H); 7.20 (d, J = 1.2 Hz, 2H); 4.04 (m, 4H); 2.64 (d, J = 6.7 Hz, 4H); 1.82-1.64 (m, 6H); 1.46-1.10 (m, 88H); 0.87 (t, J = 6.2 Hz, 18H). ^{13}C-NMR (100 MHz, $CDCl_3$, δ): 161.31; 143.77; 139.83; 136.72; 129.28; 126.64; 107.40; 42.19; 38.79; 34.81; 33.23; 33.21; 31.93; 31.91; 31.89; 30.04; 29.96; 29.72; 29.64; 29.63; 29.58; 26.50; 26.48; 22.69; 14.12. (Note: several peaks in the 13C-NMR spectrum overlap). MALDI-TOF-MS: [M^+] calc: 1084.88 found: 1084.90.

2,5-Didodecyl-3,6-bis(4-(2-ethylhexyl)thiophen-2-yl)-2,5-dihydropyrrolo[3,4-c]pyrrole-1,4-dione (7b)

The same procedure as for **7a** was followed, using **6b** (0.508 g, 9.68 × 10^{-4} mol), finely crushed potassium carbonate (K_2CO_3) powder (0.401 g, 2.90 mmol), and DMF (4 mL). After 15 min. at 120 °C, 1-bromododecane (0.724 g, 2.90 mmol) was added. The pure product (438 mg, yield: 53%) of was obtained as purple flakes after recrystallization in ethanol. ^{1}H-NMR (400 MHz, $CDCl_3$, δ): 8.71 (s, 2H); 7.21 (s, 2H); 4.04 (m, 4H); 2.65 (d, J = 6.9 Hz, 4H); 1.80-1.62 (m, 6H); 1.46-1.20 (m, 56H); 0.97-0.82 (m, 18H). ^{13}C-NMR (100 MHz, $CDCl_3$, δ): 161.32; 143.74; 139.84; 136.70; 129.31; 126.63; 107.43; 40.12; 34.38; 32.37; 31.89; 29.94; 29.61; 29.55; 29.32; 29.26; 28.76; 26.86; 25.50; 23.06; 22.67; 14.10; 10.71. MALDI-TOF-MS: [M^+] calc: 860.63 found: 860.63.

2,5-Bis(2-ethylhexyl)-3,6-bis(4-(2-ethylhexyl)thiophen-2-yl)-2,5-dihydropyrrolo[3,4-c]pyrrole-1,4-dione (7c)

The same procedure as for **7a** was followed, using **6b** (0.508 g, 9.68 × 10^{-4} mol), finely crushed potassium carbonate (K_2CO_3) powder (0.401 g, 2.90 mmol), and DMF (4 mL). After 15 min. at 120 ºC, 0.561 g (2.90 mmol) of 2-ethylhexylbromide was added. The pure product (125 mg, yield: 18%) was obtained as a waxy red solid after recrystallization in ethanol. ^{1}H-NMR (400 MHz, $CDCl_3$, δ): 8.62 (d, J = 1.0 Hz, 2H); 7.19 (s, 2H); 4.04-3.97 (m, 4H); 2.64 (d, J = 7.0 Hz, 4H); 1.91-1.81 (m, 2H); 1.71-1.61 (m, 2H); 1.38-1.18 (m, 32H); 0.92-0.82 (m, 24H). ^{13}C-NMR (100 MHz, CDCl3, δ): 161.73; 143.48; 140.19; 136.48; 129.40; 126.49; 107.72; 45.73; 40.14; 40.12; 38.97; 34.35; 32.37; 32.34; 30.15; 30.13; 28.79; 28.77; 28.31; 25.54; 25.52; 23.52; 23.06; 23.05; 14.13; 14.02; 10.75; 10.54. (Note: several peaks in the 13C-NMR spectrum overlap). MALDI-TOF-MS: [M+] calc: 748.50 found: 748.51.

3,6-Bis(5-bromo-4-(2-hexyldecyl)thiophen-2-yl)-2,5-didodecyl-2,5-dihydropyrrolo[3,4-c]pyrrole-1,4-dione (8a)

Compound **7a** (94 mg, 8.66 ×10^{-5} mol) was dissolved in chloroform (2 mL) in a dried Schlenk tube. The solution was cooled to 0 °C in the dark under stirring. After addition of

recrystallized NBS (32 mg, 1.82 $\times 10^{-4}$ mol) of in few portions, the reaction mixture was allowed to warm to room temperature. The reaction was followed by TLC and quenched with water after one hour. The organic phase was washed with water and dried over magnesium sulfate. Subsequently the solvent was evaporated *in vacuo.* The crude solid was purified further by recrystallization in ethanol with a small amount of toluene and dried overnight inside the vacuum oven. Monomer **8a** was obtained as a purple powder (77 mg, yield: 72%). 1H-NMR (200 MHz, CDCl3, δ): 8.55 (s, 2H); 3.96 (m, 4H); 2.58 (d, J = 7.2 Hz, 4H); 1.83-1.63 (m, 6H); 1.44-1.12 (m, 88H); 0.93-0.80 (m, 18H). 13C-NMR (100 MHz, CDCl3, δ): 161.00; 143.32; 138.88; 136.03; 129.00; 116.83; 107.61; 38.37; 34.12; 33.31; 33.27; 31.93; 31.91; 31.88; 30.01; 29.98; 29.69; 29.65; 29.64; 29.61; 29.58; 29.54; 29.35; 29.25; 26.84; 26.44; 26.42; 22.69; 14.13. (Note: several peaks in the 13C-NMR spectrum overlap). MALDI-TOF-MS: [M+] calc: 1240.70 found: 1240.71.

3,6-Bis(5-bromo-4-(2-ethylhexyl)thiophen-2-yl)- 2,5-didodecyl-2,5-dihydropyrrolo[3,4-c]pyrrole-1,4-dione (8b)

The same procedure as for compound **8a** was used. Now compound **7b (**400 mg, 4.64 $\times 10^{-4}$ mol) was dissolved in chloroform (9 mL) and recrystallized NBS (174 mg, 9.75 $\times 10^{-4}$ mol) was added in few portions Monomer **8b** was obtained as a purple powder (422 mg, yield: 89%). ^{1}H-NMR (400 MHz, $CDCl_3$, *δ*): 8.56 (s, 2H); 3.97 (m, 4H); 2.59 (d, *J* = 7.2 Hz, 4H); 1.80-1.64 (m, 6H); 1.46-1.16 (m, 56H); 0.97-0.81 (m, 18H). 13C-NMR (100 MHz, $CDCl_3$, *δ*): 161.02; 143.28; 138.90; 136.00; 129.02; 116.83; 107.63; 39.70; 33.71; 32.40; 31.89; 29.96; 29.62; 29.55; 29.33; 29.22; 28.66; 26.80; 25.60; 23.06; 22.67; 14.10; 10.72. MALDI-TOF-MS: [M^+] calc: 1016.45 found: 1016.44.

3,6-Bis(5-bromo-4-(2-ethylhexyl)thiophen-2-yl)-2,5-bis(2-ethylhexyl)-2,5-dihydropyrrolo[3,4-c]pyrrole-1,4-dione (8c)

The same procedure as for compound **8a** was used. Now compound **2b** (125 mg, 1.67 $\times 10^{-4}$ mol) was dissolved in chloroform (4 mL) and recrystallized NBS (62 mg, 3.50 $\times 10^{-4}$ mol) was added in few portions. Monomer **8c** was obtained as a purple solid (113 mg, yield: 75%). ^{1}H-NMR (400 MHz, $CDCl_3$, *δ*): 8.47 (d, *J* = 1.5 Hz, 2H); 3.95-3.90 (m, 4H); 2.59 (d, *J* = 7.2 Hz, 4H); 1.89-1.78 (m, 2H); 1.77-1.67 (m, 2H); 1.40-1.19 (m, 32H); 0.94-0.83 (m, 24H). 13C-NMR (100 MHz, CDCl3, δ): 161.40; 143.04; 139.24; 135.83; 129.13; 116.71; 107.87; 45.85; 39.71; 39.69; 39.01; 33.68; 32.40; 32.39; 32.37; 32.36; 30.09; 30.07; 28.69; 28.66; 28.26; 28.23; 25.65; 25.64; 25.62; 25.61; 23.53; 23.04; 14.12; 14.02; 10.75; 10.74; 10.52; 10.49. (Note: several peaks in the ^{13}C-NMR spectrum overlap). MALDI-TOF-MS: [M+] calc: 904.32 found: 904.32.

D-PDDP3T-EH

Freshly recrystallized **9** (43.0 mg, 105 μmol), monomer **8a** (107 mg, 105 μmol), recrystallized triphenylphosphine (1.65 mg, 6.30 μmol), and $Pd_2(dba)_3$ (1.44 mg, 1.57 μmol) were placed inside a dried Schlenk tube and placed under argon. Toluene (1.8 mL) and DMF (0.2 mL) were added and the mixture was degassed with argon at 40 °C. The mixture was then reacted at 115 °C overnight. The viscous polymer solution was dissolved in warm $CHCl_3$ and precipitated in methanol. The polymer was dissolved in chloroform with

ethylenediaminetetraacetic acid (EDTA) and refluxed during one hour. Water was added, refluxed for one hour and subsequently the organic layer was washed with water. The organic layer was concentrated under reduced pressure and the polymer precipitated in methanol. The polymer was then subjected to Soxhlet extraction with acetone, hexane, dichloromethane, and chloroform. Finally, the purified polymer was precipitated in methanol. $CHCl_3$ fraction: 98 mg, yield: 99%. GPC (*o*-DCB, 140 °C): M_n = 39.0 kDa, M_w = 90.0 kDa, PDI = 2.31.

D-PDDP4T-EH

Freshly recrystallized **10** (33.8 mg, 68.7 μmol), **8b** (70.0 mg, 68.7 μmol), recrystallized triphenylphosphine (1.08 mg, 4.12 μmol)) and $Pd_2(dba)_3$ (0.943 mg, 1.03 μmol) were placed under Ar in a dried Schlenk tube. Toluene (1.8 mL) and DMF (0.2 mL) were added and the mixture was degassed with argon at 40 °C. The mixture was then reacted at 115 °C overnight. The viscous polymer solution was dissolved in warm 1,1,2,2-tetrachloroethane (TCE) and precipitated in methanol. The polymer was dissolved in chloroform with ethylenediaminetetraacetic acid (EDTA) and refluxed during one hour. Water was added, refluxed for one hour and subsequently the organic layer was washed with water. The organic layer was concentrated under reduced pressure and the polymer precipitated in methanol. The precipitated polymer was then subjected to Soxhlet extraction with acetone, hexane, dichloromethane and chloroform. Swollen polymer remaining inside the Soxhlet thimble was dissolved in hot TCE and the solution was filtrated over a heated cellulose filter. Finally, the TCE solution was concentrated *in vacuo* and the purified polymer was precipitated in acetone. TCE-fraction: 54 mg, yield: 77%. GPC (*o*-DCB, 140 °C): M_n= 24.3 kDa, M_w = 59.3 kDa, PDI = 2.44. (Note: the polymer was not completely dissolved upon filtration).

D-PDDP4T-HD

The same procedure as for the polymerization of **D-PDDP4T-EH** was used, with **10** (27.7 mg, 56.3 μmol), **8a** (70.0 mg, 56.3 μmol), recrystallized PPh_3 (0.886 mg, 3.38 μmol) and $Pd_2(dba)_3$ (0.773 mg, 0.844 μmol). TCE- fraction: 60 mg, yield: 85%. GPC (o-DCB, 140 °C): M_n = 57.3 kDa, M_w = 173 kDa, PDI = 3.02.

EH-PDDP4T-EH

The same procedure as for the polymerization of **D-PDDP4T-EH** was used, with **10** (38.0 mg, 77.2 μmol), **8c** (70.0 mg, 77.2 μmol), recrystallized PPh_3 (1.22 mg, 4.63 μmol), and $Pd_2(dba)_3$ (1.06 mg, 1.16 μmol). TCE-fraction: 57 mg, yield: 81%. The molecular weight distribution of EH-PDPP4T-EH could not be determined by GPC because it has a very low solubility in o-DCB even at 140 °C.

4. UV-vis-NIR absorption spectra of the polymers in solution and film

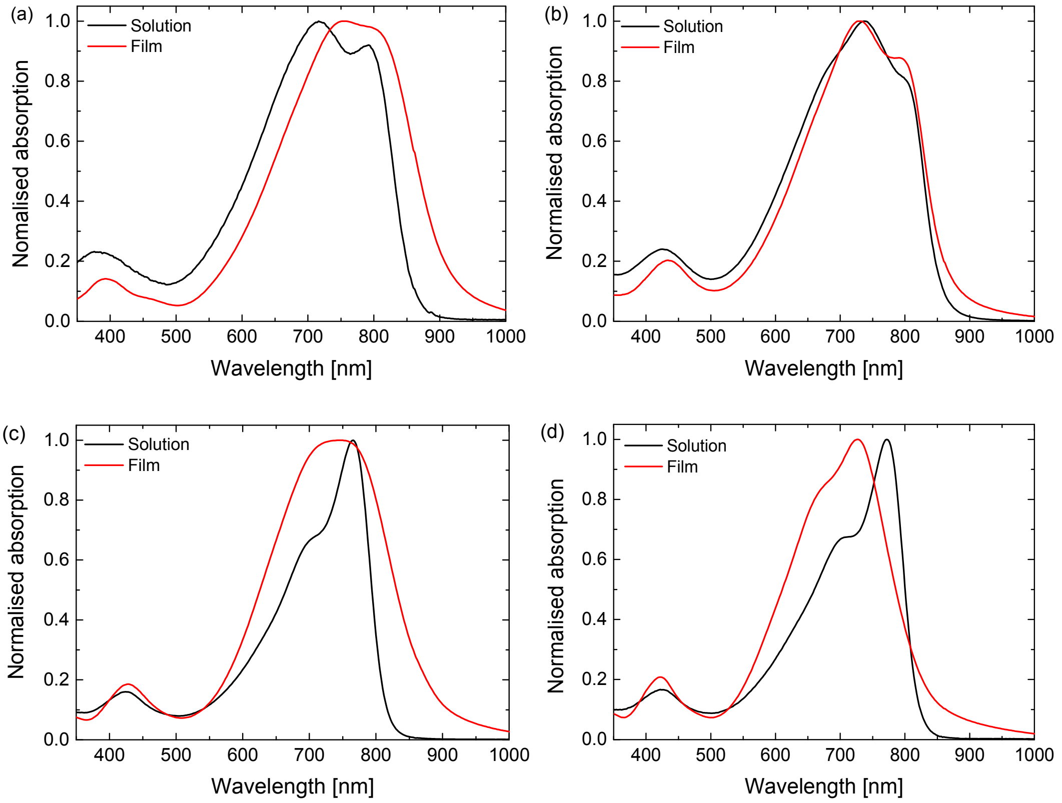

Fig. S1. UV-vis-NIR spectra of (a) D-PDPP3T-EH, (b) D-PDPP4T-EH, (c) D-PDPP4T-HD and (d) EH-PDPP4T-EH in chloroform solution and in film.

5. UV-vis-NIR absorption spectra of related PDPP3T and PDPP4T polymers in solution and film

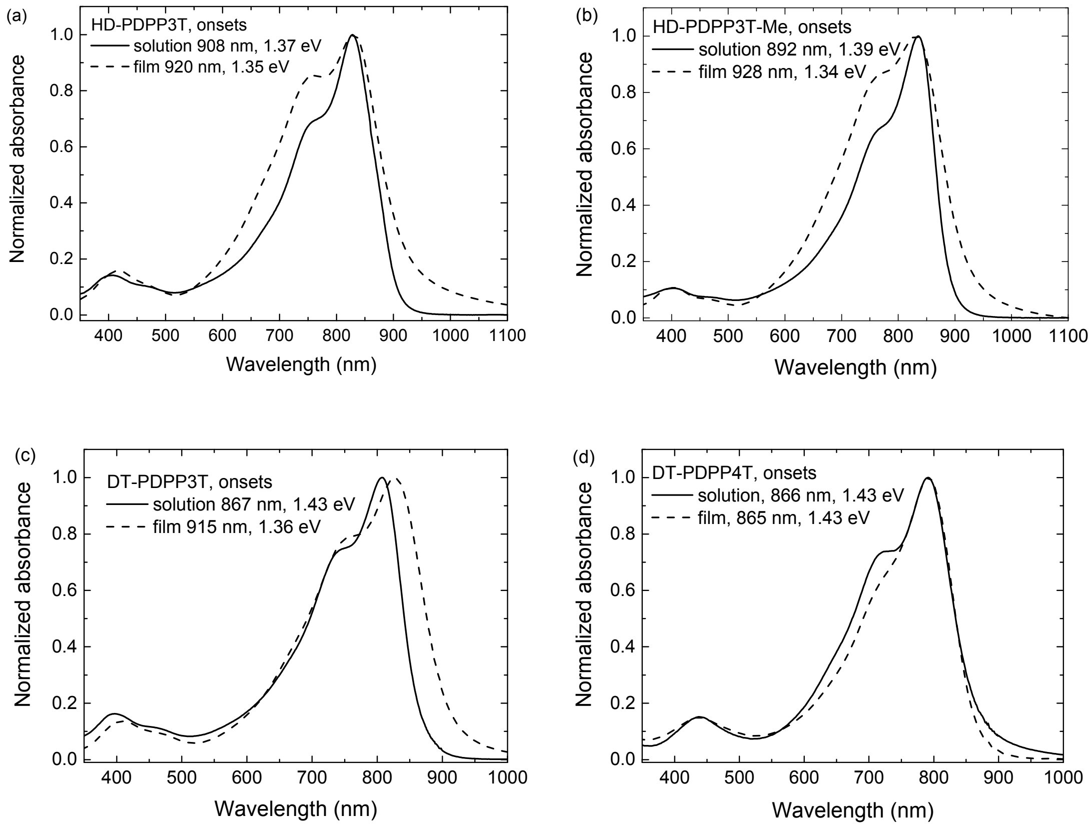

Fig. S2. UV-vis-NIR spectra of (a) HD-PDPP3T in solution and film,[S1] the onset shifts 12 nm. (b) HD-PDPP3T-Me in solution and film,[S2] the onset shifts 36 nm. (c) DT-PDPP3T in solution and film, the onset shifts 48 nm. (d) DT-PDPP4T in solution and film, the onset shifts -1 nm.[S3]

6. Cyclic voltammetry

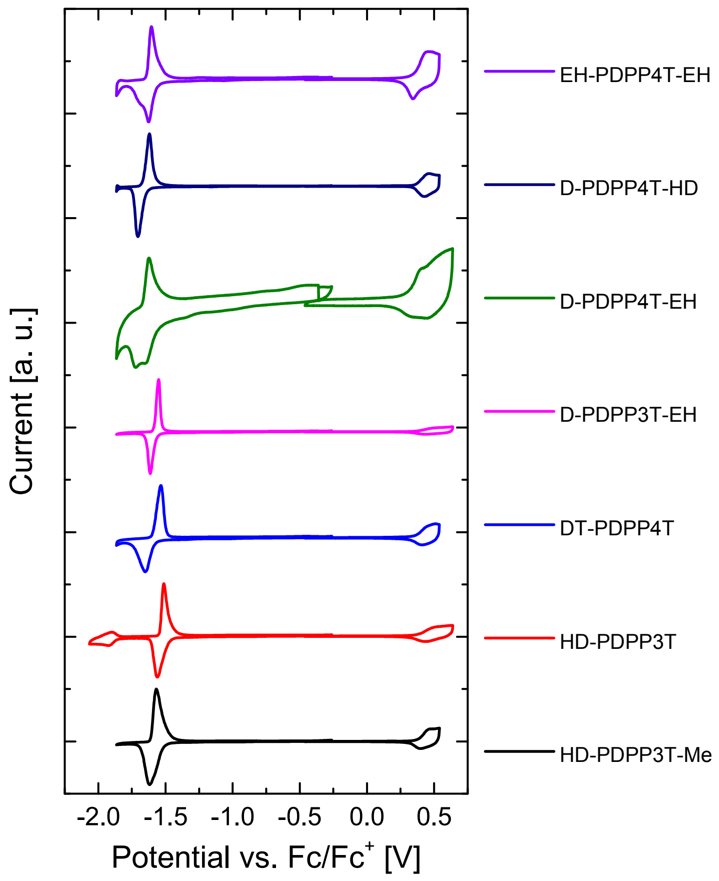

Fig. S3. Cyclic voltammograms of the polymers investigated in this work as thin films on an ITO substrate in an acetonitrile solution containing tetrabutylammonium hexafluorophosphate. The potential is referenced against ferrocene/ferrocenium.

Table S1. Onsets of the oxidation and reduction waves vs. Fc/Fc^+ and HOMO and LUMO levels determined using a level of −5.23 eV for Fc/Fc^+.

Polymer	Oxidation [V]	Reduction [V]	HOMO [eV]	LUMO [eV]
HD-PDPP3T	0.374	−1.475	−5.60	−3.76
HD-PDPP3T-Me	0.380	−1.516	−5.61	−3.71
D-PDPP3T-EH	0.366	−1.567	−5.60	−3.66
DT-PDPP4T	0.360	−1.581	−5.59	−3.65
D-PDPP4T-EH	0.312	−1.539	−5.54	−3.69
D-PDPP4T-HD	0.361	−1.646	−5.59	−3.58
EH-PDPP4T-EH	0.349	−1.581	−5.58	−3.65

7. UV-vis-NIR measurements in *o*-dichlorobenzene (*o*-OCB)

UV-vis-NIR spectra were recorded for the four polymers in *o*-OCB at different temperatures (Figure S3). *o*-OCB is a less good solvent for these polymers than chloroform or TCE. With increasing temperature of *o*-OCB D-PDPP3T-EH, D-PDPP4T-EH, and D-PDPP4T-HD show a more or less gradual loss of the long wavelength peak and its vibronic structure. At ~60 °C D-PDPP3T-EH, D-PDPP4T-HD, reach a state in which the polymer chains are truly molecularly dissolved. For D-PDPP4T-EH a small shoulder around 800 nm remained until 100 °C, which agrees with the lower solubility of this material. For EH-PDPP4T-EH the results (Figure S3d) were different from the other three polymers. At room temperature the material is sparsely soluble in *o*-DCB and when conducting the experiment from high to low temperature the polymer precipitated in its aggregated state in the cuvette, resulting in a loss of signal at lower temperatures.

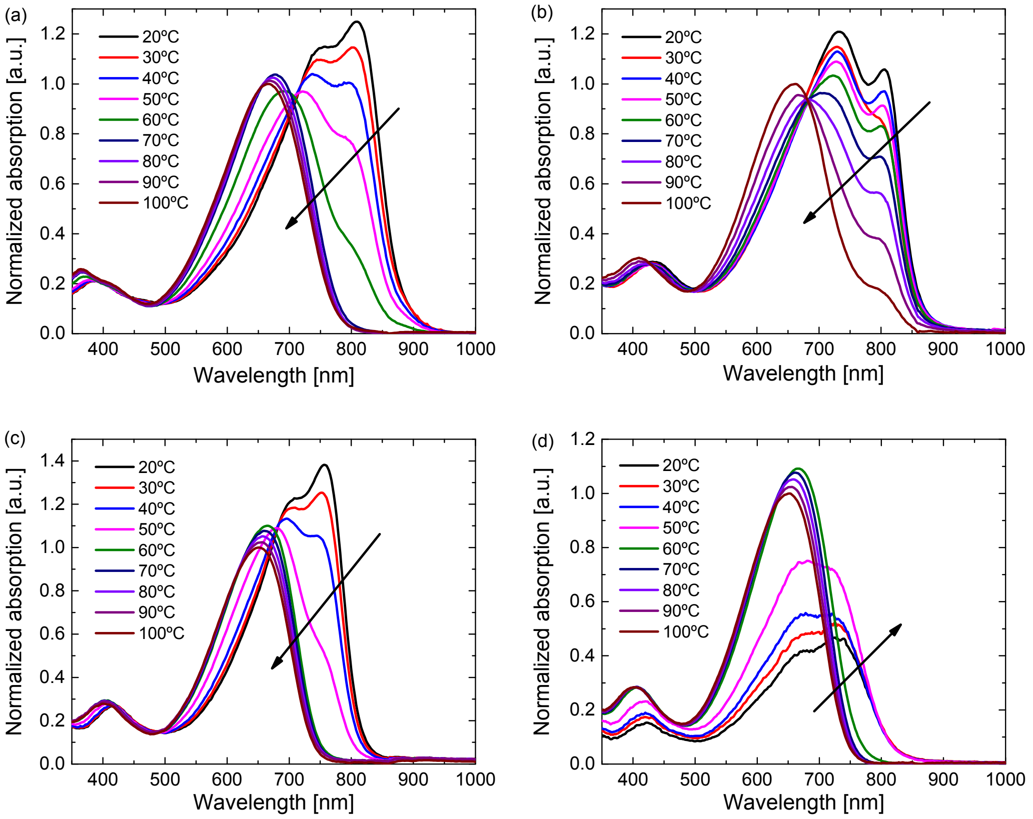

Fig. S4. The temperature dependent UV-vis-NIR absorption of the polymers in *o*-DCB solution. (a) D-PDPP3T-EH. (b) D-PDPP4T-EH. (c) D-PDPP4T-HD. (d) EH-PDPP4T-EH.

8. Space charge limited current in hole-only devices

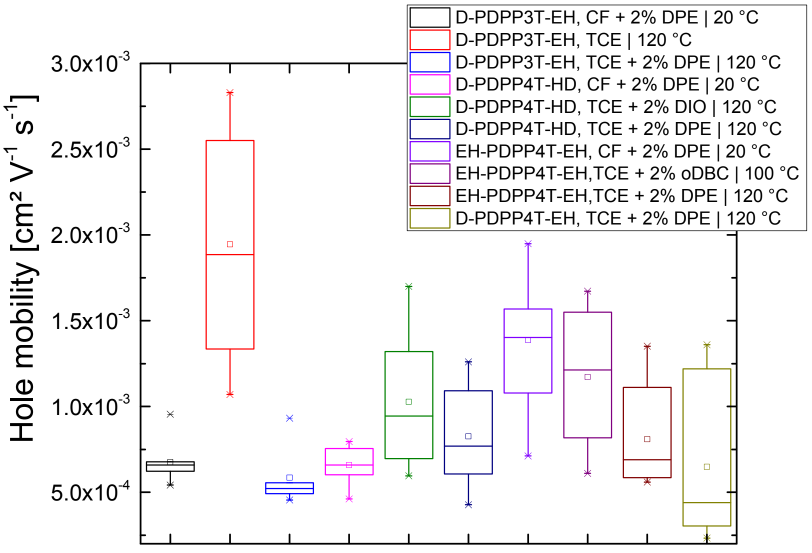

Fig. S5. Box plot of the mobility data obtained of hole only devices of the blends of the polymers with [70]PCBM under different processing conditions. The box plot and the average hole mobility reported in Table 2 of the main text are based on 12 individual devices using three different active layer thicknesses.

9. Optimization of co-solvents for solar cell devices from chloroform processed at 20 °C

Table S2. Device characteristics of the best devices for R_2-PDPP-R_1:[70]PCBM solar cells processed at 20 °C from chloroform with different co-solvents.

Polymer	**Conditions**	J_{sc}[a] [mA cm^{-2}]	V_{oc} [V]	**FF**	**PCE**[b] [%]
D-PDPP3T-EH	CF/DIO (98:2)	13.0	0.55	0.56	4.0
	CF/*o*-DCB (9:1)	13.3	0.60	0.64	5.1
	CF/DPE (98:2)	15.2	0.57	0.63	5.4
D-PDPP4T-HD	CF/DIO (98:2)	13.6	0.60	0.67	5.5
	CF/*o*-DCB (9:1)	4.3	0.64	0.72	2.0
	CF/DPE (98:2)	15.8	0.60	0.67	6.3
EH-PDPP4T-EH	CF/DIO (98:2)	12.4	0.70	0.49	4.3
	CF/*o*-DCB (9:1)	15.1	0.72	0.53	5.7
	CF/DPE (98:2)	14.9	0.71	0.58	6.1

[a] The J_{sc} values were based on integration of the EQE (measured with 1 sun bias illumination) with the AM1.5G spectrum. [b] The PCE is determined from the EQE integrated J_{sc}.

10. Optimization of co-solvents for solar cell devices from TCE processed at 120 °C

Table S3. Device characteristics for the best devices for R_2-PDPP-R_1:[70]PCBM solar cells processed at 120 °C from TCE with different co-solvents.

Polymer	Conditions	J_{sc} [mA cm^{-2}]	V_{oc} [V]	**FF**	**PCE** [%]
D-PDPP3T-EH	TCE	11.3	0.70	0.55	4.4
	TCE/DIO (98 :2)	9.4	0.55	0.55	2.8
	TCE/*o*-DCB (9:1)	5.9	0.60	0.58	2.0
	TCE/DPE (98:2)	10.4	0.59	0.62	3.8
D-PDPP4T-EH	TCE	9.3	0.61	0.58	3.3
	TCE/DIO (98 :2)	9.9	0.55	0.59	3.2
	TCE/*o*-DCB (9:1)	8.1	0.61	0.60	3.0
	TCE/DPE (98:2)	10.1	0.59	0.57	3.4
D-PDPP4T-HD	TCE	6.3	0.64	0.69	2.8
	TCE/DIO (98 :2) [c]	11.9	0.61	0.70	5.1
	TCE/*o*-DCB (9:1)	3.4	0.65	0.52	1.1
	TCE/DPE (98:2)	12.1	0.63	0.60	4.5
EH-PDPP4T-EH	TCE	11.9	0.73	0.49	4.2
	TCE/DIO (98 :2)	10.8	0.73	0.56	4.4
	TCE/*o*-DCB (9:1) [d]	10.1	0.75	0.66	5.0
	TCE/DPE (98:2)	11.4	0.74	0.59	4.9

[a] The J_{sc} values were based on integration of the EQE (measured with 1 sun bias illumination) with the AM1.5G spectrum. [b] The PCE is determined from the EQE integrated J_{sc}. [c] At 100 °C. [d] At 140 °C.

11. Hot processed cells from chloroform solutions

Table S4. Device characteristics of the best devices for R_2-PDPP-R_1:[70]PCBM solar cells processed from chloroform/DPE (98:2) at different temperatures.

Polymer	T [°C]	J_{sc}[a] [mA cm^{-2}]	V_{oc} [V]	FF	PCE [b] [%]
D-PDPP3T-EH	20	15.3 (14.8)	0.56 (0.55)	0.51 (0.48)	4.4 (3.9)
	40	15.8 (15.6)	0.56 (0.56)	0.54 (0.52)	4.8 (4.6)
	60	13.9 (13.6)	0.56 (0.56)	0.50 (0.49)	3.9 (3.7)
D-PDPP4T-HD	20	13.3 (13.1)	0.60 (0.60)	0.55 (0.54)	4.4 (4.2)
	40	12.7 (12.3)	0.60 (0.60)	0.53 (0.51)	4.1 (3.8)
	60	9.0 (9.0)	0.60 (0.60)	0.60 (0.56)	3.2 (3.0)
EH-PDPP4T-EH	20	15.3 (15.1)	0.69 (0.69)	0.52 (0.51)	5.5 (5.3)
	40	15.2 (14.8)	0.70 (0.70)	0.52 (0.51)	5.6 (5.2)
	60	13.5 (13.2)	0.71 (0.70)	0.53 (0.52)	5.1 (4.8)

[a] Values between parentheses are the values for the average performance (based on 4 devices). The J_{sc} values were based on integration of the EQE (measured with 1 sun bias illumination) with the AM1.5G spectrum. [b] The PCE is determined from the EQE integrated J_{sc}. All devices were made on the same day from the same solutions.

12. Processing conditions for optimized solar cells and device statistics

Table S5. Processing conditions and best and average device characteristics for R_2-PDPP-R_1:[70]PCBM solar cells.

Polymer	Conditions	T [°C]	J_{sc} [mA cm^{-2}]	V_{oc} [V]	FF	PCE [%]
D-PDPP3T-EH	CF/DPE (98:2)	20	15.2 (15.1)	0.57 (0.57)	0.63 (0.62)	5.4 (5.3)
D-PDPP3T-EH	TCE	120	11.3 (11.0)	0.70 (0.69)	0.55 (0.55)	4.4 (4.3)
D-PDPP3T-EH	TCE/DPE (98:2)	120	10.4 (10.6)	0.59 (0.58)	0.62 (0.59)	3.8 (3.6)
D-PDPP4T-EH	TCE/DPE (98/2)	120	10.1 (10.0)	0.59 (0.58)	0.57 (0.56)	3.4 (3.3)
D-PDPP4T-HD	CF/DPE (98:2)	20	15.8 (15.4)	0.60 (0.60)	0.67 (0.66)	6.3 (6.1)
D-PDPP4T-HD	TCE/DIO (98:2)	100	11.9 (11.9)	0.61 (0.61)	0.70 (0.68)	5.1 (4.9)
D-PDPP4T-HD	TCE/DPE (98/2)	120	12.1 (11.7)	0.63 (0.63)	0.60 (0.60)	4.5 (4.4)
EH-PDPP4T-EH	CF/DPE (98:2)	20	14.9 (14.8)	0.71 (0.71)	0.58 (0.58)	6.1 (5.9)
EH-PDPP4T-EH	TCE/*o*-DCB (9:1)	140	10.1 (10.0)	0.75 (0.74)	0.66 (0.65)	5.0 (4.9)
EH-PDPP4T-EH	TCE/DPE (98/2)	120	11.4 (11.1)	0.74 (0.73)	0.59 (0.57)	4.9 (4.6)

[a] Values between parentheses are the values for the average performance (based on 4 devices). The J_{sc} values were based on integration of the EQE (measured with 1 sun bias illumination) with the AM1.5G spectrum. [b] The PCE is determined from the EQE integrated J_{sc}.

References

S1 K. H. Hendriks, G. H. L. Heintges, V. S. Gevaerts, M. M. Wienk and R. A. J. Janssen, *Angew. Chem. Int. Ed.*, 2013, **52**, 8341–8344.

S2 W. Li, A. Furlan, K. H. Hendriks, M. M. Wienk and R. A. J. Janssen, *J. Am. Chem. Soc.*, 2013, **135**, 5529–5532.

S3 W. Li, K. H. Hendriks, A. Furlan, W. S. C. Roelofs, M. M. Wienk and R. A. J. Janssen, *J. Am. Chem. Soc.*, 2013, **135**, 18942−18948.
